# Impact of Malperfusion Burden on Early Outcomes After Surgery for Type A Acute Aortic Dissection: A Retrospective, Single-Center Investigation

**DOI:** 10.3390/jcm15082999

**Published:** 2026-04-15

**Authors:** Matteo Marro, Gustavo Alfredo Sobrino Avellaneda, Domitilla Di Lorenzo, Andrea De Laurentis, Francesca Panvini, Andrea Costamagna, Marco Pocar, Michele William La Torre, Massimo Boffini, Antonio Loforte, Mauro Rinaldi

**Affiliations:** 1 Cardiac Surgery Division, Department of Surgical Sciences, AOU Città della Salute e della Scienza di Torino, University of Turin, 10126 Turin, Italy; 2 Anesthesiology and Intensive Care Division, Department of Surgical Sciences, AOU Città della Salute e della Scienza di Torino, University of Turin, 10126 Turin, Italy

**Keywords:** acute type A aortic dissection, malperfusion burden, malperfusion patterns, early outcomes, perioperative risk

## Abstract

**Objectives:** Malperfusion is a major determinant of outcome in acute type A aortic dissection (ATAAD), yet its heterogeneous patterns and prognostic impact remain incompletely defined. We investigated the association between malperfusion burden, territory-specific involvement, and early outcomes after emergency ATAAD repair. **Methods:** We performed a retrospective single-center study including 483 consecutive patients undergoing emergency surgery for ATAAD (2010–2022). Malperfusion was classified by coronary, visceral, and peripheral territories and stratified as none, single-territory, or multidistrict (≥2 territories). The primary outcome was in-hospital mortality. Secondary outcomes included stroke, renal replacement therapy, peri-procedural myocardial infarction, major vascular events, and a composite endpoint of major adverse events (MAEs). Multivariable logistic regression identified independent predictors. **Results:** Overall, 68.5% of the population were male with a mean age of 65.4 ± 12.1 years. Malperfusion was present in 151 patients (31.3%), including 131 (27.1%) with single-territory and 20 (4.1%) with multidistrict involvement. In-hospital mortality increased stepwise with malperfusion burden (12.7%, 19.8%, and 50.0%; *p* < 0.001). MAEs occurred in 36.6% of patients, with a similar gradient (31.2%, 46.2%, and 65.0%, *p* < 0.001). In multivariable analysis, preoperative shock, neurological deficit, descending aortic involvement, and redo surgery were independent predictors of MAEs, whereas malperfusion burden showed an attenuated association after adjustment. Territory-specific analyses revealed strong associations between coronary malperfusion and peri-procedural myocardial infarction, visceral malperfusion and postoperative dialysis, and peripheral malperfusion and major vascular events. **Conclusions:** Malperfusion burden is associated with worse early outcomes after ATAAD repair but largely reflects underlying clinical severity. Distinct malperfusion territories confer specific postoperative risks, supporting a pattern-based approach to perioperative risk stratification.

## 1. Introduction

Acute type A aortic dissection (ATAAD) remains one of the most lethal cardiovascular emergencies, with in-hospital mortality rates still ranging between 15% and 25% despite advances in surgical techniques, perioperative management, and critical care [1]. Emergency surgical repair represents the cornerstone of treatment and has significantly improved survival compared with medical management alone. However, early outcomes remain highly variable, reflecting the heterogeneity of patient presentation and disease severity at admission.

Among preoperative risk factors, malperfusion syndrome (MPS) has consistently been identified as one of the strongest predictors of adverse outcomes [2]. MPS is a devastating sequela of ATAAD that is characterized by end-organ dysfunction secondary to ischemia, arising from dissection flaps that cause static or dynamic arterial obstruction of aortic branch vessels. MPS may affect coronary, cerebral, visceral, renal, and peripheral vascular territories, leading to myocardial ischemia, neurological injury, metabolic derangement, and multiorgan failure. These pathophysiological consequences often complicate perioperative management and contribute substantially to early morbidity and mortality. Large international registries and contemporary cohort studies have clearly demonstrated that the presence of malperfusion is independently associated with increased early and late mortality after surgical repair of ATAAD [3,4,5,6]. Moreover, recent investigations have suggested a graded relationship between the number of malperfused vascular territories and clinical outcomes, indicating that malperfusion burden may influence prognosis in a dose-dependent manner [7,8,9,10].

The severity, anatomical distribution, and combination of affected vascular territories may differ markedly among patients, potentially resulting in heterogeneous prognostic profiles. Nevertheless, most available evidence has primarily focused on malperfusion as a global risk marker rather than as a heterogeneous clinical entity. In particular, while previous investigations have provided important insights into the prognostic role of malperfusion, the clinical relevance of specific malperfusion patterns and their combined effects on early surgical outcomes remains an area of ongoing investigation. As a result, the prognostic implications of specific malperfusion configurations have not yet been fully clarified. Whether isolated coronary, visceral, or peripheral malperfusion carries comparable risk, and how multidistrict involvement influences early postoperative morbidity and mortality, remains an important area of investigation in contemporary surgical cohorts managed with standardized institutional protocols. Therefore, the present study aimed to investigate the impact of distinct malperfusion patterns on early outcomes in patients undergoing emergency surgery for ATAAD. By adopting a pattern-based analytical approach within a large single-center experience, we sought to provide a more refined and clinically actionable risk stratification framework to support perioperative decision-making and individualized patient management.

## 2. Methods

### 2.1. Study Design and Patient Population

This study was designed as a retrospective observational cohort analysis conducted at a single tertiary referral center for aortic surgery. All consecutive adult patients who underwent emergency surgical repair for ATAAD between January 2010 and December 2022 were screened for eligibility. ATAAD was defined according to current international guidelines as a dissection involving the ascending aorta with symptom onset within 14 days prior to presentation [11]. Diagnosis was established by contrast-enhanced computed tomography (CT) angiography in all patients. Patients were included if they underwent primary surgical repair for ATAAD during the study period. Exclusion criteria were: chronic dissections, intramural hematoma without intimal tear, traumatic dissections, iatrogenic dissections, patients who died before surgical intervention, and those managed non-operatively. Clinical, imaging, operative, and postoperative data were prospectively collected in an institutional aortic surgery database and retrospectively reviewed for the purposes of the present study. The study protocol was approved by the local institutional review board, and the requirement for individual informed consent was waived owing to the retrospective nature of the study and the use of anonymized data.

### 2.2. Definition

Malperfusion was defined as evidence of impaired blood flow to an aortic branch vessel attributable to dissection-related static, dynamic, or combined obstruction, as documented by preoperative clinical assessment, laboratory findings, contrast-enhanced CT scan, and/or intraoperative findings. Malperfusion was considered present when at least one of the following criteria was met: (1) radiographic evidence of reduced or absent branch-vessel perfusion, (2) clinical signs of end-organ ischemia, or (3) laboratory abnormalities consistent with organ hypoperfusion. MPS was defined as malperfusion associated with objective evidence of end-organ dysfunction, including metabolic derangement, renal impairment, visceral ischemia, myocardial ischemia, or neurological deficit. Malperfusion was categorized according to the affected vascular territory as follows:

- Coronary malperfusion, defined by clinical, electrocardiographic, biochemical, and/or intraoperative evidence of myocardial ischemia attributable to dissection-related coronary involvement.
- Visceral malperfusion, defined by radiographic evidence of compromised flow to mesenteric and/or celiac vessels and/or clinical and laboratory findings consistent with visceral ischemia.
- Peripheral malperfusion, defined by clinical signs of limb ischemia, including pulse deficit, pain, or neurological impairment, with radiographic confirmation when available.

Classification was based on integrated preoperative and intraoperative assessment and institutional database records.

### 2.3. Study Outcomes

The primary outcome was in-hospital mortality, defined as death occurring during the index hospitalization for surgical repair of ATAAD. Secondary outcomes included major postoperative complications occurring during the index hospitalization, specifically:

- Stroke, defined as a new focal or global neurological deficit persisting >24 h, with radiological confirmation when available.
- Renal failure requiring renal replacement therapy (RRT), defined as the need for postoperative dialysis (intermittent hemodialysis and/or continuous renal replacement therapy).
- Peri-procedural acute myocardial infarction (AMI), defined as a postoperative myocardial ischemic event supported by a compatible clinical and/or electrocardiographic pattern and an elevation of cardiac biomarkers, with additional confirmatory evidence when available (new regional wall motion abnormality on echocardiography and/or angiographic evidence of coronary compromise).
- Major vascular events, defined as the occurrence of acute limb ischemia requiring surgical or endovascular revascularization and/or fasciotomy and/or mesenteric ischemia requiring laparotomy and/or bowel resection.

A composite endpoint of major adverse events (MAEs) was additionally evaluated, defined as the occurrence of any of the following: in-hospital death, stroke, dialysis, periprocedural AMI or major vascular events.

### 2.4. Statistical Analysis

Continuous variables are reported as mean ± standard deviation or median (interquartile range), as appropriate based on data distribution. Categorical variables are presented as counts and percentages. Between-group comparisons were performed using Student’s *t*-test or the Mann–Whitney *U* test for continuous variables, and the χ^2^ test or Fisher’s exact test for categorical variables, as appropriate. For the primary analysis, patients were stratified according to malperfusion burden into three groups: no malperfusion, single-territory malperfusion, and multidistrict malperfusion (≥2 vascular territories). In secondary analyses, malperfusion was also evaluated according to specific vascular territories (coronary, visceral, and peripheral). Associations between malperfusion patterns and binary outcomes—including in-hospital mortality, postoperative stroke, renal failure requiring RRT, peri-procedural AMI, MVEs, and major adverse events—were initially assessed using univariable logistic regression. Multivariable logistic regression was subsequently performed to identify independent predictors of the composite endpoint. Covariates were selected a priori based on clinical relevance and included age, chronic kidney disease, preoperative neurological deficit, preoperative shock, preoperative resuscitation, descending aortic involvement, redo cardiac surgery, and malperfusion pattern. Model parsimony was ensured by limiting the number of covariates relative to the number of outcome events.

The primary composite endpoint included in-hospital death (including intraoperative mortality), postoperative stroke, renal failure requiring dialysis, peri-procedural myocardial infarction, and major vascular events. A sensitivity analysis was performed among operative survivors, defining a postoperative composite endpoint including stroke, need for dialysis, peri-procedural AMI, MVEs, and in-hospital death occurring after surgery.

In territory-specific analyses, the associations between coronary, visceral, and peripheral malperfusion and selected outcomes were evaluated using logistic regression, with additional minimal adjustment for age and preoperative shock.

Results are reported as odds ratios (ORs) with 95% confidence intervals (CIs). All statistical tests were two-sided, and a *p*-value < 0.05 was considered statistically significant. Statistical analyses were performed using JASP 0.95.4 (Amsterdam, The Netherlands).

## 3. Results

A total of 483 consecutive patients undergoing emergency surgery for ATAAD were included in the present analysis. Based on the assessment of coronary, visceral, and peripheral vascular territories, any malperfusion was identified in 151 patients (31.3%). Malperfusion involved a single vascular territory in 131 patients (27.1%), whereas multidistrict malperfusion (≥2 territories) was observed in 20 patients (4.1%). The remaining 332 patients (68.7%) had no documented malperfusion. Baseline characteristics, intraoperative variables, and early postoperative outcomes stratified according to malperfusion pattern are summarized in Table 1. When stratified by vascular territory, coronary malperfusion was observed in 57 patients (11.8%), visceral malperfusion in 43 patients (8.9%), and peripheral malperfusion in 71 patients (14.7%). Isolated coronary, visceral, and peripheral malperfusion occurred in 51 (10.6%), 27 (5.6%), and 53 (11.0%) patients, respectively. Overall, in-hospital mortality was 16.1%, occurring in 42 of 332 (12.7%) patients without malperfusion, in 26 of 131 patients (19.8%) with single-territory malperfusion, and in 10 of 20 patients (50.0%) with multidistrict malperfusion. In twenty patients (4.1%), postoperative outcomes could not be evaluated because of intraoperative death.

Postoperative stroke (fatal or non-fatal) occurred in 57 patients (12.3% of the patients who survived surgery), including 11 fatal strokes, 32 strokes with residual disability, and 14 strokes without disability. Transient ischemic attack was observed in 3 patients (0.6%). Among patients with postoperative stroke, neurological events occurred within 24 h in 37 patients (64.9%) and within 30 days in 19 patients (33.3%), indicating that most events developed in the early perioperative period. Preoperative neurological deficits were present in 61 patients (12.6%); among patients who developed postoperative stroke, 14 (24.6%) had pre-existing neurological impairment at presentation, whereas 43 (75.4%) had no documented neurological deficit before surgery.

Postoperative RRT was required in 82 patients (17.7%), including 69 patients (14.9%) who required temporary dialysis and 13 patients (2.8%) who remained dialysis-dependent at discharge. Preoperative chronic kidney disease (estimated glomerular filtration rate < 60 mL/min/1.73 m^2^) was present in 89 patients (18.4%) and was associated with a higher incidence of postoperative dialysis (25.8% vs. 15.0% in patients without chronic kidney disease). In addition, three patients (0.6%) were receiving dialysis preoperatively, all of whom required postoperative renal replacement therapy. Overall, peri-procedural AMI occurred in 9 patients (1.9%), including 6 of 57 patients (10.5%) with preoperative coronary malperfusion or ECG evidence of infarction; among the 9 patients, 5 (55.5%) underwent concomitant coronary artery bypass grafting during the ATAAD surgery.

MVEs occurred in 40 patients (8.6%). When stratified according to preoperative visceral and/or peripheral malperfusion, the incidence of MVEs was higher among patients with visceral and/or peripheral malperfusion (15, 15.5%) compared with those without visceral or peripheral malperfusion (25, 6.9%). Among the total MVEs, 6 (15%) patients required exploratory laparotomy.

**Figure 1:**
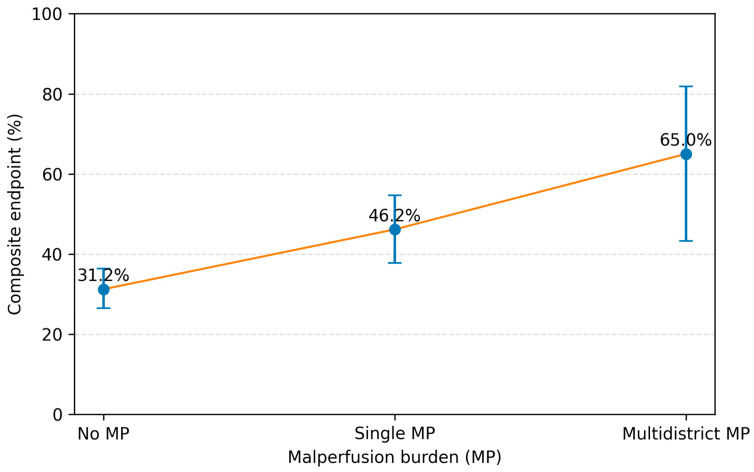
Stepwise increase in the composite endpoint according to malperfusion burden.

**Figure 2:**
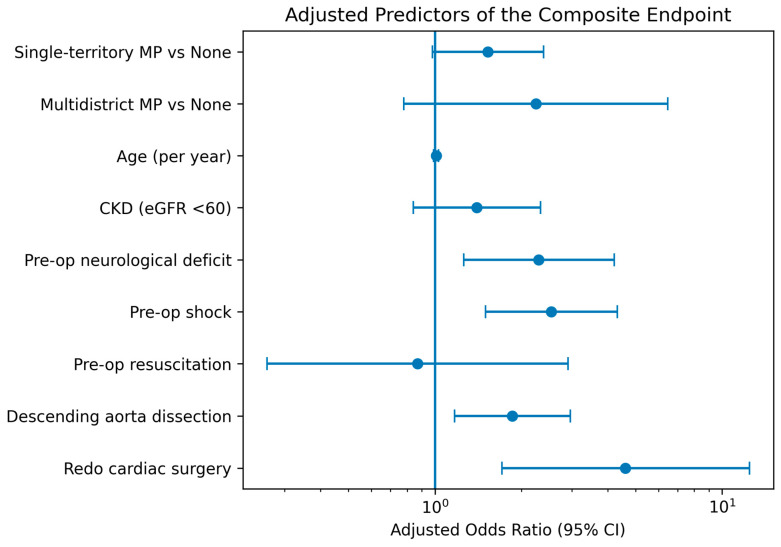
Forest plot showing adjusted odds ratios and 95% confidence intervals for independent predictors of the composite endpoint in multivariable logistic regression analysis. The vertical line indicates an odds ratio of 1.0. MP, malperfusion; CKD, chronic kidney disease.

The composite endpoint occurred in 177 of 483 patients (36.6%). The incidence increased stepwise with malperfusion burden, occurring in 104 of 333 patients (31.2%) without malperfusion, 60 of 130 (46.2%) with single-territory malperfusion, and 13 of 20 (65.0%) with multidistrict malperfusion (*p* < 0.001) (Figure 1). In univariable logistic regression, single-territory malperfusion (OR 1.89, 95% CI 1.25–2.86; *p* = 0.0027) and multidistrict malperfusion (OR 4.09, 95% CI 1.59–10.55; *p* = 0.0036) were associated with higher odds of the composite endpoint (overall *p* < 0.001). In multivariable logistic regression analysis, pre-operative shock (adjusted OR 2.54, 95% CI 1.50–4.32; *p* < 0.001), pre-operative neurological deficit (adjusted OR 2.30, 95% CI 1.26–4.21; *p* = 0.0069), descending aortic involvement (adjusted OR 1.86, 95% CI 1.17–2.96; *p* = 0.009), and redo cardiac surgery (adjusted OR 4.61, 95% CI 1.71–12.45; *p* = 0.0026) were independently associated with the composite endpoint. After adjustment for these preoperative factors, malperfusion pattern showed an attenuated association with the composite endpoint (single-territory vs. none: adjusted OR 1.53, 95% CI 0.98–2.39; *p* = 0.062; multidistrict vs. none: adjusted OR 2.25, 95% CI 0.78–6.47; *p* = 0.134; overall *p* = 0.089), Table 2. Independent predictors of the composite endpoint are illustrated in Figure 2. These findings were consistent in sensitivity analyses restricted to operative survivors, with a similar stepwise increase in the incidence of the composite endpoint across malperfusion categories, Table 3.

In territory-specific analyses performed among operative survivors, preoperative coronary malperfusion was strongly associated with peri-procedural myocardial infarction (11.3% vs. 0.7%; unadjusted OR 17.28, 95% CI 4.18–71.36; *p* < 0.001), and this association persisted after minimal adjustment for age and preoperative shock (adjusted OR 16.97, 95% CI 4.07–70.73; *p* < 0.001). Visceral malperfusion was associated with a higher incidence of postoperative dialysis (31.0% vs. 16.4%; adjusted OR 2.27, 95% CI 1.12–4.62; *p* = 0.023). Peripheral malperfusion showed a borderline association with postoperative dialysis (26.1% vs. 16.3%; adjusted OR 1.80, 95% CI 0.98–3.29; *p* = 0.057). For major vascular events, peripheral malperfusion was strongly associated with increased risk (20.3% vs. 6.6%; adjusted OR 3.65, 95% CI 1.78–7.45; *p* < 0.001), whereas visceral malperfusion was not (9.5% vs. 8.6%; adjusted OR 1.07, 95% CI 0.36–3.19; *p* = 0.91), Table 4.

## 4. Discussion

The present study provides a comprehensive, single-center evaluation of the impact of malperfusion burden and territory-specific malperfusion on early outcomes after emergency surgery for acute type A aortic dissection. Several important findings emerge. First, malperfusion was common, affecting nearly one-third of patients, and was associated with a clear stepwise increase in early mortality and major adverse events. Second, after adjustment for preoperative clinical severity, the prognostic impact of malperfusion burden was attenuated, suggesting that malperfusion primarily reflects the severity of systemic compromise at presentation rather than acting as an isolated risk factor. Third, territory-specific analyses revealed distinct and biologically plausible associations between individual malperfusion territories and specific postoperative complications, highlighting the heterogeneity of malperfusion as a clinical entity.

Patients with increasing malperfusion burden also presented with progressively higher clinical severity, including a higher prevalence of preoperative shock, renal dysfunction, and neurological impairment. In addition, they more frequently required complex surgical strategies, including extensive arch repair, frozen elephant trunk implantation, and concomitant coronary revascularization, with longer cardiopulmonary bypass times. These baseline and procedural differences likely contributed to the attenuation of the independent effect of malperfusion after multivariable adjustment and underscore the close interrelationship between anatomical malperfusion and systemic disease severity.

Large contemporary registries and multicenter studies have consistently demonstrated that malperfusion syndrome is among the strongest predictors of adverse outcomes in ATAAD. Data from IRAD, GERAADA, the Nordic Consortium, and recent nationwide analyses have shown markedly increased early mortality in patients presenting with malperfusion, particularly when multiple vascular territories are involved [4,5,12,13,14,15,16]. Our findings are concordant with these observations, confirming a graded relationship between malperfusion burden and early adverse outcomes, with multidistrict malperfusion conferring the highest risk. Importantly, however, our multivariable analysis demonstrates that the association between malperfusion burden and the composite endpoint is substantially attenuated after adjustment for preoperative shock, neurological deficit, and other markers of disease severity. This observation aligns with prior registry data suggesting that malperfusion often coexists with profound hemodynamic instability and end-organ dysfunction, which themselves are powerful determinants of outcome [17]. In this context, malperfusion appears less as an isolated prognostic factor and more as a marker of advanced systemic compromise at presentation. This interpretation is consistent with analyses from IRAD and GERAADA, in which adjustment for clinical severity reduced—but did not eliminate—the effect of malperfusion on early mortality [3,5].

A key contribution of the present study is the detailed evaluation of territory-specific malperfusion and its differential impact on postoperative complications. While previous studies have largely treated malperfusion as a binary variable or focused primarily on overall mortality, our analyses demonstrate that different vascular territories are associated with distinct postoperative risk profiles. Coronary malperfusion was strongly and independently associated with peri-procedural myocardial infarction, even after minimal adjustment for age and shock. This finding is clinically intuitive and reinforces the importance of heightened myocardial protection strategies and careful coronary assessment in this high-risk subgroup, as also suggested by prior IRAD and single-center studies [10]. Visceral malperfusion was associated with a significantly increased risk of postoperative renal replacement therapy, reflecting the close pathophysiological relationship between visceral ischemia, metabolic derangement, and acute kidney injury. Interestingly, visceral malperfusion was not independently associated with major vascular events as defined in this study, suggesting that its primary impact may be mediated through systemic metabolic and inflammatory pathways rather than through discrete vascular complications. Peripheral malperfusion emerged as a particularly strong predictor of major vascular events, with more than a threefold increased risk. This finding supports the concept that peripheral malperfusion may act as a marker of diffuse systemic ischemia and extensive dissection burden, a notion increasingly emphasized in the contemporary literature and recent guideline documents [8,9,18,19]. The borderline association between peripheral malperfusion and postoperative dialysis further underscores its role as an indicator of global hypoperfusion rather than isolated limb ischemia. These findings have several important clinical implications. First, they support a more nuanced risk stratification strategy that goes beyond the simple presence or absence of malperfusion and incorporates both malperfusion burden and territorial involvement. Second, the attenuation of malperfusion’s effect after adjustment highlights the critical importance of early recognition and aggressive management of shock and end-organ dysfunction at presentation. Third, territory-specific associations may help guide perioperative decision-making, resource allocation, and postoperative surveillance, particularly in patients with coronary, visceral, or peripheral malperfusion. In line with recent guideline recommendations and emerging concepts in the management of complex ATAAD, our data support an individualized approach that integrates anatomical findings with clinical severity rather than relying on malperfusion as a uniform prognostic label.

Recent technological advancements, including artificial intelligence (AI) and Internet of Things (IoT)-based systems, are increasingly shaping the field of vascular and aortic surgery. In the context of acute aortic syndromes, AI-driven imaging analysis has shown promise in improving the rapid detection of aortic dissection and in identifying subtle signs of branch-vessel malperfusion on computed tomography scans. Furthermore, predictive analytics models integrating clinical, imaging, and laboratory data may enhance early risk stratification and support decision-making in critically ill patients [20]. In parallel, IoT-enabled monitoring systems allow continuous real-time acquisition of physiological parameters in both preoperative and postoperative settings, facilitating early detection of hemodynamic instability and end-organ dysfunction [21]. In complex aortic surgery, such as ATAAD repair, these technologies may contribute to more timely recognition of malperfusion-related complications and enable more personalized perioperative management strategies. Although these approaches are still evolving and not yet routinely integrated into clinical practice, they represent a promising avenue for improving outcomes in this high-risk population.

In addition, three-dimensional (3D) printing is emerging as a valuable adjunct in vascular and aortic surgery, bridging the gap between imaging, education, and clinical practice. Patient-specific 3D models derived from computed tomography data allow detailed visualization of complex aortic anatomy, including the extent of dissection and branch-vessel involvement. In the setting of chronic thoracic aortic dissection, such models may facilitate preoperative planning, particularly in cases with complex arch anatomy or multiterritory malperfusion, by improving spatial understanding and aiding in the selection of surgical strategies. Furthermore, 3D printing has demonstrated significant value in surgical training and simulation, allowing surgeons to rehearse procedures and better anticipate technical challenges. Although its routine clinical application remains limited, 3D printing represents a promising tool for integrating advanced imaging into personalized surgical planning and education in complex aortic disease [22].

## 5. Limitations

This study has several limitations. Its retrospective, single-center design may limit generalizability, and unmeasured confounding cannot be excluded despite multivariable adjustment. Malperfusion was defined based on clinical, laboratory, and imaging findings and did not distinguish between dynamic and static mechanisms. Long-term outcomes were not assessed, and the analysis focused exclusively on early in-hospital events. Nevertheless, the large sample size, detailed characterization of malperfusion patterns, and robust sensitivity analyses strengthen the validity of our findings.

## 6. Conclusions

In patients undergoing emergency surgery for ATAAD, malperfusion burden is associated with worse early outcomes, but its prognostic impact is closely intertwined with the severity of preoperative clinical presentation. Territory-specific malperfusion patterns confer distinct risks for specific postoperative complications, underscoring the heterogeneous nature of malperfusion syndromes. These findings support a more refined, pattern-based approach to risk stratification and highlight the need to address systemic instability and end-organ dysfunction as key determinants of early outcome.

## Figures and Tables

**Table 1:** Baseline, intraoperative variables, and early outcomes according to malperfusion pattern.

Variable	Overall (n = 483)	No MP (n = 332)	Single MP (n = 131)	Multi MP (n = 20)	*p*
***Preoperative characteristics***					
Age, years	65.4 ± 12.1	65.8 ± 11.9	64.0 ± 12.6	69.0 ± 12.1	0.132
Female sex	152 (31.5%)	111 (33.4%)	34 (26.0%)	7 (35.0%)	0.278
BMI, kg/m^2^	27.4 ± 10.3	27.4 ± 12.0	27.4 ± 5.0	27.5 ± 4.1	0.153
LVEF, %	56.4 ± 6.3	56.7 ± 6.2	56.2 ± 6.1	54.0 ± 7.9	0.299
Diabetes	20 (4.1%)	17 (5.1%)	3 (2.3%)	0 (0.0%)	0.25
CKD (pre-op eGFR < 60)	89 (18.4%)	56 (16.9%)	26 (19.8%)	7 (35%)	0.045
Serum creatinine, mg/dL	1.0 (0.9–1.3)	1.0 (0.9–1.2)	1.1 (0.9–1.4)	1.2 (0.9–1.4)	0.003
***Comorbidities***					
Diabetes	46 (9.5%)	33 (9.9%)	12 (9.2%)	1 (5.0%)	0.781
COPD	26 (5.4%)	22 (6.6%)	4 (3.1%)	0 (0.0%)	0.167
Redo cardiac surgery	16 (3.3%)	8 (2.4%)	7 (5.3%)	1 (5.0%)	0.173
***Presentation***					
Preoperative shock	85 (17.6%)	41 (12.3%)	32 (24.4%)	12 (60.0%)	<0.001
CPR before surgery	13 (2.7%)	6 (1.8%)	4 (3.1%)	3 (15.0%)	0.018
Preoperative neurological deficit	61 (12.6%)	37 (11.1%)	19 (14.5%)	5 (25.0%)	0.080
Descending aorta dissection	327 (67.7%)	204 (61.4%)	103 (786.2%)	20 (100.0%)	<0.001
***Intraoperative variables***					
Aortic cross-clamp time, min	86 (67–112)	84 (65–110)	90 (70–116)	98 (79–130)	0.070
CPB time, min	177 (140–226)	172 (137–221)	186 (146–235)	211 (168–259)	0.02
Antegrade cerebral perfusion (unilateral/bilateral)	442 (91.5%)	301 (90.7%)	122 (93.1%)	19 (95.0%)	0.36
Root procedure	58 (12%)	35 (10.5%)	20 (15.3%)	3 (15.0%)	0.19
Arch replacement	47 (9.7%)	31 (9.3%)	11 (8.4%)	5 (20.0%)	0.028
Frozen elephant trunk	16 (3.3%)	7 (2.1%)	6 (4.6%)	3 (15.0%)	0.006
Concomitant CABG	30 (6.2%)	12 (3.6%)	15 (11.5%)	3 (15.0%)	<0.001
***Early postoperative outcomes***					
In-hospital mortality	78 (16.1%)	42 (12.7%)	26 (19.8%)	10 (50.0%)	<0.001
Stroke	57 (11.8%)	37 (11.1%)	17 (13.0%)	3 (15.0%)	0.716
Dialysis	82 (17.0%)	56 (16.9%)	23 (17.6%)	3 (15.0%)	0.974
AMI	9 (1.9%)	3 (0.9%)	6 (4.6%)	0 (0.0%)	0.020
MVEs	40 (8.3%)	21 (6.3%)	16 (12.2%)	3 (15.0%)	0.006
Re-exploration for bleeding	41 (8.5%)	24 (7.2%)	15 (11.5%)	2 (10.0%)	0.229
Red Blood Cell Transfusion (n)	4 (1–7)	4 (1–6)	4 (1–8)	6 (3–9.5)	0.02

BMI: Body Mass Index; CKD: Chronic Kidney Disease; COPD: Chronic Obstructive Pulmonary Disease; LVEF: Left Ventricle Ejection Fraction; CPR: Cardio Pulmonary Resuscitation; CABG: Coronary Artery Bypass Grafting; CPB: Cardio Pulmonary Bypass; AMI: Acute Myocardial Infarction; MVEs: Major Vascular Events.

**Table 2:** Multivariable logistic regression analysis for independent predictors of the composite endpoint.

Variable	Adjusted OR	95% CI	*p*
**Malperfusion pattern**			
Single vs. none	1.53	0.98–2.39	0.062
Multidistrict vs. none	2.25	0.78–6.47	0.134
Age (per year)	1.01	0.99–1.03	0.37
CKD (eGFR < 60)	1.40	0.84–2.33	0.199
Pre-op neurological deficit	2.30	1.26–4.21	0.0069
Pre-op shock	2.54	1.50–4.32	<0.001
Resuscitation pre-op	0.87	0.26–2.91	0.825
Descending aorta dissection	1.86	1.17–2.96	0.009
Redo cardiac surgery	4.61	1.71–12.45	0.0026

CKD: Chronic Kidney Disease; eGFR: Estimated Glomerular Filtration Rate.

**Table 3:** Primary and Sensitivity Analyses of Malperfusion Burden and Composite Outcomes.

Analysis	Population	Composite Definition	None n/N (%)	Single n/N (%)	Multi n/N (%)	OR Single vs. None (95% CI)	OR Multi vs. None (95% CI)	Global *p*
**Primary analysis**	All patients (n = 483)	In-hospital death, Stroke, Dialysis, AMI, MVEs	104/333 (31.2)	60/130 (46.2)	13/20 (65.0)	1.89 (1.25–2.86)	4.09 (1.59–10.55)	0.00037
**Sensitivity analysis**	Operative survivors (n = 463)	Post-op death Stroke, Dialysis, AMI, MVEs	90/319 (28.2)	54/124 (43.5)	13/20 (65.0)	1.96 (1.28–3.02)	4.73 (1.83–12.23)	0.00014

AMI: Acute Myocardial Infarction; MVEs: Major Vascular Events; OR: Odds Ratio; CI: confidence interval.

**Table 4:** Territory-Specific Associations Between Preoperative Malperfusion and Postoperative Outcomes.

Preoperative Malperfusion	Outcome	Unadjusted OR (95% CI)	*p*	Adjusted OR * (95% CI)	*p*
**Coronary**	AMI	17.28 (4.18–71.36)	<0.001	16.97 (4.07–70.73)	<0.001
**Visceral**	Post-op dialysis	2.28 (1.13–4.61)	0.022	2.27 (1.12–4.62)	0.023
**Peripheral**	Post-op dialysis	1.81 (1.00–3.31)	0.052	1.80 (0.98–3.29)	0.057
**Visceral**	MVEs	1.12 (0.38–3.32)	0.83	1.07 (0.36–3.19)	0.91
**Peripheral**	MVEs	3.59 (1.77–7.30)	<0.001	3.65 (1.78–7.45)	<0.001

* Adjusted for age and preoperative shock. AMI: Acute Myocardial Infarction; MVEs: Major Vascular Events; OR: Odd-Ratio; CI: confidence interval.

## Data Availability

The original contributions presented in this study are included in the article. Further inquiries can be directed to the corresponding author.
